# SGII: Systematic Identification of Essential lncRNAs in Mouse and Human Genome With lncRNA-Protein-Protein Heterogeneous Interaction Network

**DOI:** 10.3389/fgene.2022.864564

**Published:** 2022-03-21

**Authors:** Xiao-Hong Xin, Ying-Ying Zhang, Chu-Qiao Gao, Hui Min, Likun Wang, Pu-Feng Du

**Affiliations:** ^1^ College of Intelligence and Computing, Tianjin University, Tianjin, China; ^2^ Institute of Systems Biomedicine, Department of Pathology, School of Basic Medical Sciences, Beijing Key Laboratory of Tumor Systems Biology, Peking-Tsinghua Center of Life Sciences, Peking University Health Science Center, Beijing, China

**Keywords:** essential lncRNA, lncRNA-protein interaction network, protein-protein interaction network, network centrality, systematic method

## Abstract

Long noncoding RNAs (lncRNAs) play important roles in a variety of biological processes. Knocking out or knocking down some lncRNA genes can lead to death or infertility. These lncRNAs are called essential lncRNAs. Identifying the essential lncRNA is of importance for complex disease diagnosis and treatments. However, experimental methods for identifying essential lncRNAs are always costly and time consuming. Therefore, computational methods can be considered as an alternative approach. We propose a method to identify essential lncRNAs by combining network centrality measures and lncRNA sequence information. By constructing a lncRNA-protein-protein interaction network, we measure the essentiality of lncRNAs from their role in the network and their sequence together. We name our method as the systematic gene importance index (SGII). As far as we can tell, this is the first attempt to identify essential lncRNAs by combining sequence and network information together. The results of our method indicated that essential lncRNAs have similar roles in the LPPI network as the essential coding genes in the PPI network. Another encouraging observation is that the network information can significantly boost the predictive performance of sequence-based method. All source code and dataset of SGII have been deposited in a GitHub repository (https://github.com/ninglolo/SGII).

## Introduction

Long noncoding RNAs (lncRNAs) refer to non-coding RNAs with a length over 200 nt. LncRNAs play a major role in epigenetic control, cell differentiation, autophagy, apoptosis, and embryonic development (Mercer et al., 2009; Rinn and Chang, 2012; Chen, 2016). Many cellular processes are regulated by lncRNAs. For examples, RNA splicing, translation, and signal transductions are related to lncRNA regulations (Khalil and Rinn, 2011; Da Sacco et al., 2012; Zhu et al., 2013; Hu et al., 2017; Zhang et al., 2018, 2021; Li et al., 2019; Pyfrom et al., 2019; Zhao et al., 2020). In addition, lncRNAs are related to a variety of complex diseases, including cancers, nervous system diseases, and cardiovascular diseases (Fenoglio et al., 2013; Uchida and Dimmeler, 2015; Schmitt and Chang, 2016).

Knocking out or knocking down some lncRNA genes can lead to death or infertility. These lncRNAs are called essential lncRNAs, which are of vital importance for survival and development. Identification of essential lncRNAs provides insight into the minimum requirements of normal cell functioning and normal organism development. Experimental methods have been applied to identify essential lncRNAs. Li *et al.* established the single lncRNA knockout mouse model, as well as the multiple lncRNA knockout mouse model (Li and Chang, 2014). By large-scale phenotypic analysis, they found that knocking out lncRNAs, such as *Fendrr*, *Peril*, and *Mdgt*, showed perinatal and postpartum lethality (Li and Chang, 2014). Watanabe *et al.* found that *Dnm3os* has an essential role in the normal growth and bone development of mice (Watanabe et al., 2008). Zhou *et al.* proposed that *Meg3* deletion in female rats can result in skeletal muscle defect and perinatal death (Zhou et al., 2012). These studies provide helpful insights for identifying essential lncRNAs. However, experimental methods for identifying essential lncRNA genes are not always feasible due to many factors, which may also produce misleading results (Jathar et al., 2017). Therefore, computational approaches are considered as alternative ways.

Computing essentiality of a coding gene has been widely studied. Most of the existing methods define the essentiality measures based on the topological importance of a protein in protein-protein interaction networks. Various types of centralities have been introduced in this regard. For example, Jeong *et al.* found that hub nodes with high connections in the protein-protein interaction (PPI) network are often indispensable, which allows them to use the degree centrality (DC) to identify essential proteins (Jeong et al., 2001). Joey *et al.* introduced the betweenness centrality (BC) to measure the essentiality of proteins, as they found that PPI network is modularized (Joy et al., 2005). Wang *et al.* used eigenvector centrality (EC) to predict essential proteins, which measures the importance of nodes by calculating the connection with high index nodes in the network (Wang et al., 2013). Wuchty *et al.* found that closeness centrality (CC) measure using local information is useful in predicting essential proteins (Wuchty and Stadler, 2003). Many more methods have tried to incorporate different types of information in predicting essential proteins (Li et al., 2012; Wang et al., 2012; Zhong et al., 2013; Campos et al., 2019; Zhang et al., 2020; Liu et al., 2021). However, these centrality measures are not always working, due to incomplete protein-protein networks and frequent false-positives in the high-throughput experiments for identifying protein-protein interactions. Therefore, sequence-based methods were also considered. Zeng *et al.* defined the Gene Importance Calculator (GIC) score using only genomic sequence information (Zeng et al., 2018). The GIC score was derived from a logistic regression model. It can score not only coding genes but also non-coding genes.

As far as we can tell, the GIC score is the only available essentiality measure that can be applied on non-coding genes, including lncRNA genes (Zeng et al., 2018). However, the design of the GIC score ignored all information that is buried in the lncRNA-protein interactions (LPI). We believe that the LPI information has a similar role in identifying essential lncRNAs to that of PPI in identifying essential coding genes.

With the development of high-throughput experimental technologies, many databases have been established for non-coding genes and their interactions. The NPInter database provides a comprehensive archive of molecular interactions involving noncoding RNAs(Hao et al., 2016). NONCODE database is an integrated knowledge database dedicated to non-coding RNAs and their annotations (Zhao et al., 2016). However, essential gene databases, like the DEG database, focus more on recording essential coding genes (Zhang et al., 2004). The essential non-coding genes are rarely recorded, particularly for complex organisms, like human and mouse. This is a primary challenge in developing a systematic method for measuring essentiality of non-coding genes.

By curating data from various literatures, as well as public databases, we established a dataset as the basis for developing a computational method to measure non-coding gene essentiality. In this work, we proposed the systematic gene importance index (SGII) by combining various centralities on the lncRNA-protein-protein heterogeneous network and sequence-based essentiality scores. By comparing our measure to both network-based methods and sequence-based method, we found that network information can boost the sequence-based method significantly.

## Materials and Methods

### Dataset Curation

We downloaded human and mouse lncRNA-protein interactions from the NPInter database v4.0 (Hao et al., 2016). Self-interactions and duplicates were removed. The mouse lncRNA-protein interaction network involves 33255 lncRNAs, 182 proteins, and 102051 interactions. The human lncRNA-protein interaction network contains 41589 lncRNAs, 3237 proteins, and 394895 interactions. We downloaded human and mouse protein-protein interaction data from BioGrid database version 4.4 (Oughtred et al., 2021). The mouse protein-protein interaction network includes 9744 proteins and 52342 interactions. The human protein-protein interaction network includes 19106 proteins and 644235 interactions.

We combine the lncRNA-protein interactions and protein-protein interactions by matching the name of the proteins in both datasets, producing a heterogeneous network with two types of interactions. The mouse network was composed by 9845 proteins and 33255 lncRNAs with 102051 lncRNA-protein interactions and 52342 protein-protein interactions. The human network was composed by 19553 proteins and 41589 lncRNAs with 394895 lncRNA-protein interactions and 644235 protein-protein interactions. The sequences of all lncRNAs in both human and mouse interaction networks were obtained from the NONCODE database version 5 (Zhao et al., 2016).

According to literatures (Penny et al., 1996; Marahrens et al., 1997; Lee, 2000; Sado et al., 2001; Grote et al., 2013; Klattenhoff et al., 2013; Sauvageau et al., 2013; Yildirim et al., 2013; Zeng et al., 2018), eight mouse lncRNAs, including *Xist*, *Gas*5, *Meg*3, *Tsix*, *Gt* (ROSA) 26*Sor*, *Dnm*3*os*, *Fendrr*, and *Braveheart*, were identified as essential lncRNAs. The remaining 33247 lncRNAs in the mouse network were marked with unknown status. For human lncRNAs, we curated a set of lncRNAs that are reported to be essential in various conditions from literatures (Supplementary Table S1). This set contains 63 lncRNAs. The names of these lncRNAs and the conditions that they are reported to be essential, are listed in Supplementary Table S1, along with literatures of the original reports. In addition, 11 mouse lncRNAs, which are homologous of human essential lncRNAs, were also collected for validation purpose, as homologous usually have similar essentiality (Georgi et al., 2013).

### Gene Importance Calculator

Gene Importance Calculator (GIC) (Zeng et al., 2018) is a useful essentiality indicator for both protein-coding genes and noncoding genes. It is based solely on sequence information. The GIC score (*g*) is defined as follows:

$$g=\frac{1}{1+\exp\left[-\theta \left(p\right)\right]},$$ (1)

where *θ*(*p*) is derived from a logistic regression model. *θ*(*p*) can be defined as

$$\theta \left(p\right)=\ln\frac{p}{1-p}=\beta_{0}+\beta_{1}L+\beta_{2}\frac{1}{L}e+\sum_{i=1}^{5}\alpha_{i}f_{i},$$ (2)

where *α*_1_, *α*_2_, …, *α*_5_, *β*_0_, *β*_1_ and *β*_2_ are regression coefficients, *L* the length of RNA sequence, *e* the minimum free energy of RNA secondary structure, *p* the conditional probability that a gene is essential, and *f*_*i*_ the occurrence frequency of a triplet in the sequence. The five types of triplets, which are considered in the GIC, are CGA, GCG, TCG, ACG and TCA (Zeng et al., 2018).

When calculating the GIC score, we need to use the external program RNAfold (Lorenz et al., 2011), which requires a sequence length less than 20000 nt. Therefore, only 24450 mouse lncRNAs and 29481 human lncRNAs can be calculated for GIC. All other lncRNAs have lengths too long for the RNAfold to work.

### Network Centralities

We formulate the heterogeneous graph as ***G*** = (***V***, ***E***), where ***V*** is the set of all nodes, including lncRNAs and proteins, and ***E*** the set of all interactions, including lncRNA-protein and protein-protein interactions. Without losing generality, we note the number of all nodes as *n*. The network can be represented as an adjacency matrix **A**∈{0.1}^*n*×*n*^. The element on the *i*th row and the *j*th column of **A** can be denoted as *a*_*i*,*j*_. If *a*_*i*,*j*_ = 1, the *i*th node and the *j*th node have interactions between them. If *a*_*i*,*j*_ = 0, there is no interaction between the *i*th node and the *j*th node. Given *a*_*i*,*j*_, we can define four different centrality measures, including degree centrality (DC), betweenness centrality (BC), closeness centrality (CC), and eigenvector centrality (EC) for each node in the network.

The degree centrality of the *i*th node can be defined as follows:

$$d_{i}=\frac{1}{n-1}\sum_{j=1}^{n}a_{i,j}$$ (3)

The betweenness centrality of the *i*th node can be defined as follows:

$$b_{i}=\frac{1}{\left(n-1\right)\left(n-2\right)}\sum_{u\neq i\neq v\in V}\frac{\sigma_{u,v}\left(i\right)}{\sigma_{u,v}}$$ (4)

where *σ*_*u*,*v*_ is the number of shortest paths between the *u*th node and the *v*th node, and *σ*_*u*,*v*_(*i*) the number of shortest paths between the *u*th node and the *v*th node that pass the *i*th node.

The closeness centrality of the *i*th node is defined as follows:

$$c_{i}=\frac{{\left[\left|R\left(i\right)\right|-1\right]}^{2}}{\left(n-1\right)\sum_{j\in R\left(i\right)}d_{i,j}}$$ (5)

where ***R*** (*i*) is the set of nodes that can reach the *i*th node, *d*_*i*,*j*_ the length of the shortest path between the *i*th node and the *j*th node, and |.| cardinal operator of a set.

The eigenvector centrality of the *i*th node is defined as follows:

$$e_{i}=x_{\max}\left(i\right)$$ (6)

where *x*_max_(*i*) is the *i*th dimension of the normalized eigen vector **x** that corresponds to the largest eigen value of adjacency matrix **A**. Let *λ*_max_ be the largest eigen value of **A**, the following relationships are satisfied in finding **x**:

$$\mathrm{Ax}=\lambda_{\max}x,\;\mathrm{and}$$ (7)

‖x‖=1 (8)

where ||.|| is the vector norm operator.

### Systematic Gene Importance Index

**FIGURE 1:**
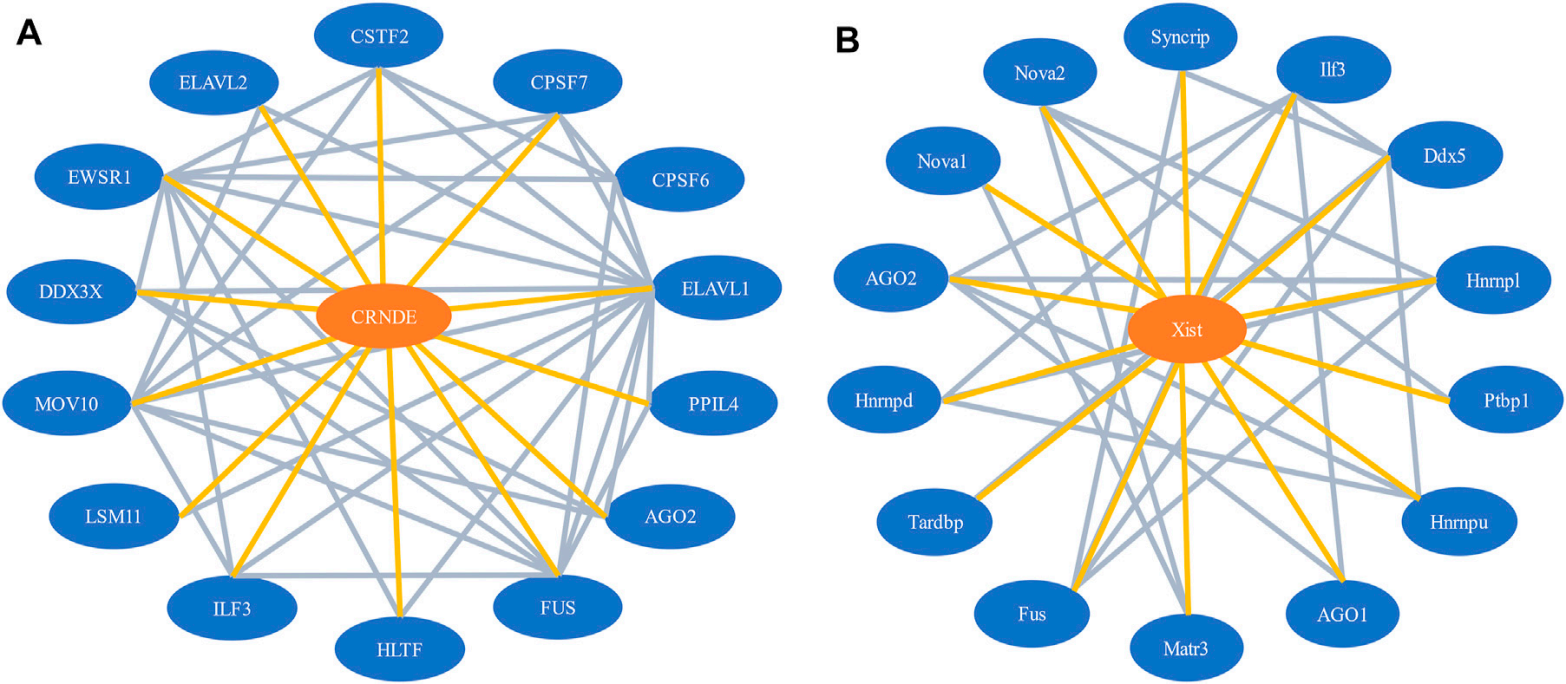
A part of the LPPI network. **(A)** Human dataset. The network contains the lncRNA *CRNDE* and 14 interacting proteins; **(B)** Mouse dataset. The network contains the lncRNA *Xist* and 14 interacting proteins.

Our network model contains two types of nodes, lncRNAs, and proteins. It also involves two types of interactions, the lncRNA-protein interactions and protein-protein interactions. Essentially, it is a lncRNA-protein-protein interaction (LPPI) heterogeneous network. Figure 1 illustrates a part of the LPPI network for human and mouse respectively.

We propose the Systematic Gene Importance Index (SGII) as a comprehensive measure of gene essentiality, particularly for non-coding genes. SGII is a combination of the sequence-based GIC score and centrality measures, which have been elaborated as above.

For the *i*th node in the LPPI network, we compute its BC, CC, DC and EC, which can be noted as *b*_*i*_, *c*_*i*_, *d*_*i*_ and *e*_*i*_, respectively. Its GIC score is noted as *g*_*i*_. We sort all nodes according to their BC, CC, DC, EC and GIC in a descending order, respectively. The rank of the *i*th node after sorting according to BC, CC, DC, EC and GIC can be noted as *r*_*b*_(*i*), *r*_*c*_(*i*), *r*_*d*_(*i*), *r*_*e*_(*i*) and *r*_*g*_(*i*), respectively.

Let *s*_*i*_ be the degree of the *i*th node, which can be computed as follows:

$$s_{i}=\sum_{j=1}^{n}a_{i,j}$$ (9)

Given a threshold *z*, if *s*_*i*_ ≥ *z*, the centrality measures will determine the essentiality of a gene directly. For convenience, we define the centrality-based essentiality indicator function for the *i*th node according to BC, CC, DC, and EC respectively as follows:

$$I_{b}\left(i\right)=\{\begin{array}{cc} 1 & \frac{r_{b}\left(i\right)}{n}<k\%,\\ 0 & otherwise \end{array}$$ (10)

$$I_{c}\left(i\right)=\{\begin{array}{cc} 1 & \frac{r_{c}\left(i\right)}{n}<k\%,\\ 0 & otherwise \end{array}$$ (11)

$$I_{d}\left(i\right)=\{\begin{array}{cc} 1 & \frac{r_{d}\left(i\right)}{n}<k\%,\\ 0 & otherwise \end{array},\text{and}$$ (12)

$$I_{e}\left(i\right)=\{\begin{array}{cc} 1 & \frac{r_{e}\left(i\right)}{n}<k\%,\\ 0 & otherwise \end{array}$$ (13)

where *k* is a rank threshold parameter. The *i*th node is identified as essential when

$$I_{b}\left(i\right)I_{c}\left(i\right)I_{d}\left(i\right)I_{e}\left(i\right)=1$$ (14)

is satisfied.

If *s*_*i*_ < *z*, we rely on the GIC score to determine the essentiality of a gene. Similarly, we can define the indicator function for GIC ranking, as follows:

$$I_{g}\left(i\right)=\{\begin{array}{cc} 1 & \frac{r_{g}\left(i\right)}{n}<t\%,\\ 0 & otherwise \end{array}$$ (15)

where *t* is another rank threshold parameter. The *i*th node is essential if

$$I_{g}\left(i\right)=1$$ (16)

is satisfied.

**FIGURE 2:**
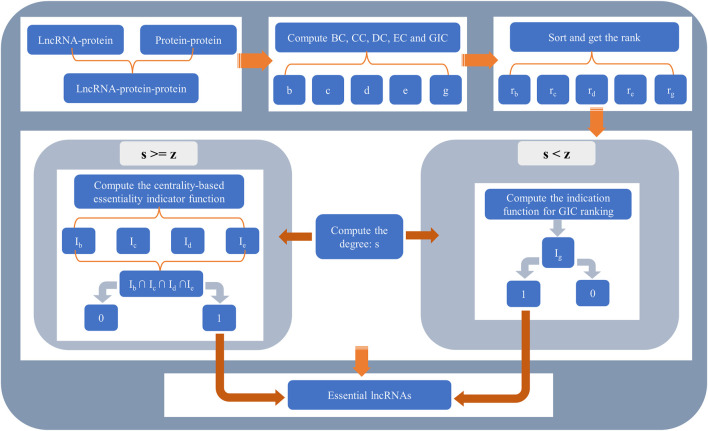
The flowchart of SGII. The method SGII consists of two parts. For lncRNAs whose degree is greater than or equal to *z*, four types of centralities were used to determine whether they were essential lncRNAs. For lncRNAs whose degree is less than *z*, GIC was used.

The whole flowchart of SGII is illustrated in Figure 2.

### Performance Evaluation

In evaluating SGII, we use three statistics to describe its predictive performance. These statistics include sensitivity (*s*), false positive rate (*r*), and Fisher’s exact test score (*f*), which are defined as follows:

$$s=\frac{n_{t}}{n_{+}}$$ (17)

$$r=\frac{n_{f}}{n_{-}},\text{and}$$ (18)

$$f=-\log_{10}p$$ (19)

where *n*_*t*_ is the number of known essential lncRNAs that are identified as essential lncRNAs, *n*_+_ the total number known essential lncRNAs, *n*_*f*_ the number of lncRNAs with unknown essentiality that are identified as essential, *n*_-_ the total number of lncRNAs with unknown essentiality and *p* the *p*-value of Fisher’s exact test. Since SGII is a direct scoring method with manually configurable cutoff values, no training procedure is involved in the whole process. This is different to machine learning based methods. We cannot treat the above sensitivity and false positive rate as comparable to those in evaluating machine learning methods, as the knowledge of essential lncRNAs is too limited to perform any kind of cross-validations. This is also why we introduced the Fisher’s exact test to further quantifying the quality of our results. It will measure how likely a result in whole is random or not. The bigger *f* value is, the results are less likely to be random.

### Parameter Calibration

There are eight parameters in the GIC, which represent all the coefficients in the model built by GIC method. We took all the parameter values from literature (Zeng et al., 2018). The values for the mouse model are *β*_0_ = 0.1625, *β*_1_ = 2.638 × 10^–4^, *β*_2_ = 2.194, *α*_1_ = 19.88 (for CGA), *α*_2_ = 37.59 (for GCG), *α*_3_ = 50.37 (for TCG), *α*_4_ = 35.44 (for ACG), and *α*_5_ = -64.66 (for TCA). The values for human model are *β*_0_ = 0.7417, *β*_1_ = 2.612 × 10^–4^, *β*_2_ = 4.295, *α*_1_ = 48.66, *α*_2_ = 15.64, *α*_3_ = 76.23, *α*_4_ = -1.113, and *α*_5_ = -60.29.

Three parameters are introduced in combining centralities and GIC, which are noted as *z*, *k* and *t*. We first perform a grid search of *k* and *t* with a given value of *z*. The pairs of *k* and *t*, which maximize the score *f*, are recorded for every different *z*. These values are further sorted to find the best *z*, *k* and *t* combination. When performing the grid search on the mouse dataset, *k* = 1, 3, 5, 7, 9, and *t* = 1, 3, 5, 7, 9. When performing the grid search on the human dataset, *k* = 5, 10, 15, 20, 25 and *t* = 5, 10, 15, 20, 25. For both datasets, *z* = 5, 10, 15, 20. Finally, we set *z* = 15, *k* = 5, *t* = 9 for mouse dataset, and *z* = 5, *k* = 20, *t* = 5 for human dataset. All results for different parameters are provided in supplementary materials, as Supplementary Table S2.

## Results and Discussions

### Characters of the lncRNA-Protein-Protein Heterogeneous Network

**FIGURE 3:**
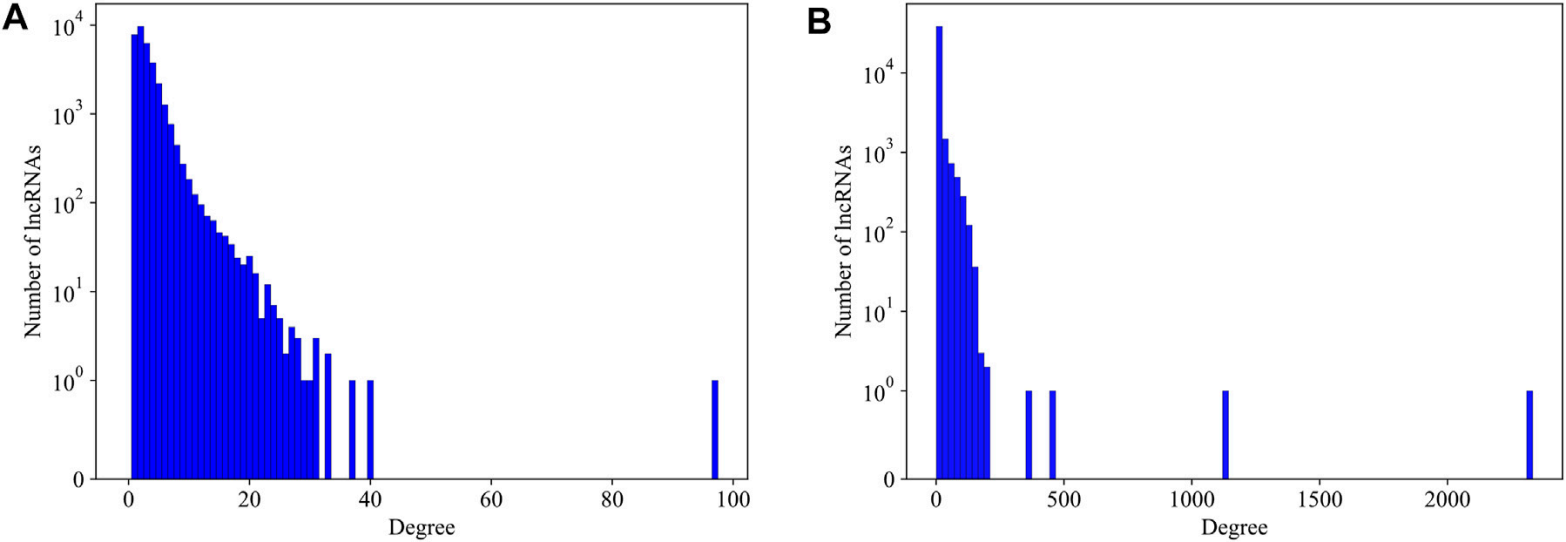
The degree distribution of lncRNAs in mouse network and human network, respectively. **(A)** The degree distribution of lncRNAs in mouse network; **(B)** The degree distribution of lncRNAs in human network. No protein-protein interaction is counted in producing these distributions.

We first explore the basic statistical characters of the LPPI network. We plot the degree distribution of the mouse and human network respectively in Figure 3. It is intuitively that the distribution of the degree follows the common power law distribution, which is similar to the PPI networks (Jeong et al., 2001). Since in the PPI network, essential proteins are usually rare and with high degrees, we assume that in our LPPI network, the essential lncRNAs have similar properties.

**FIGURE 4:**
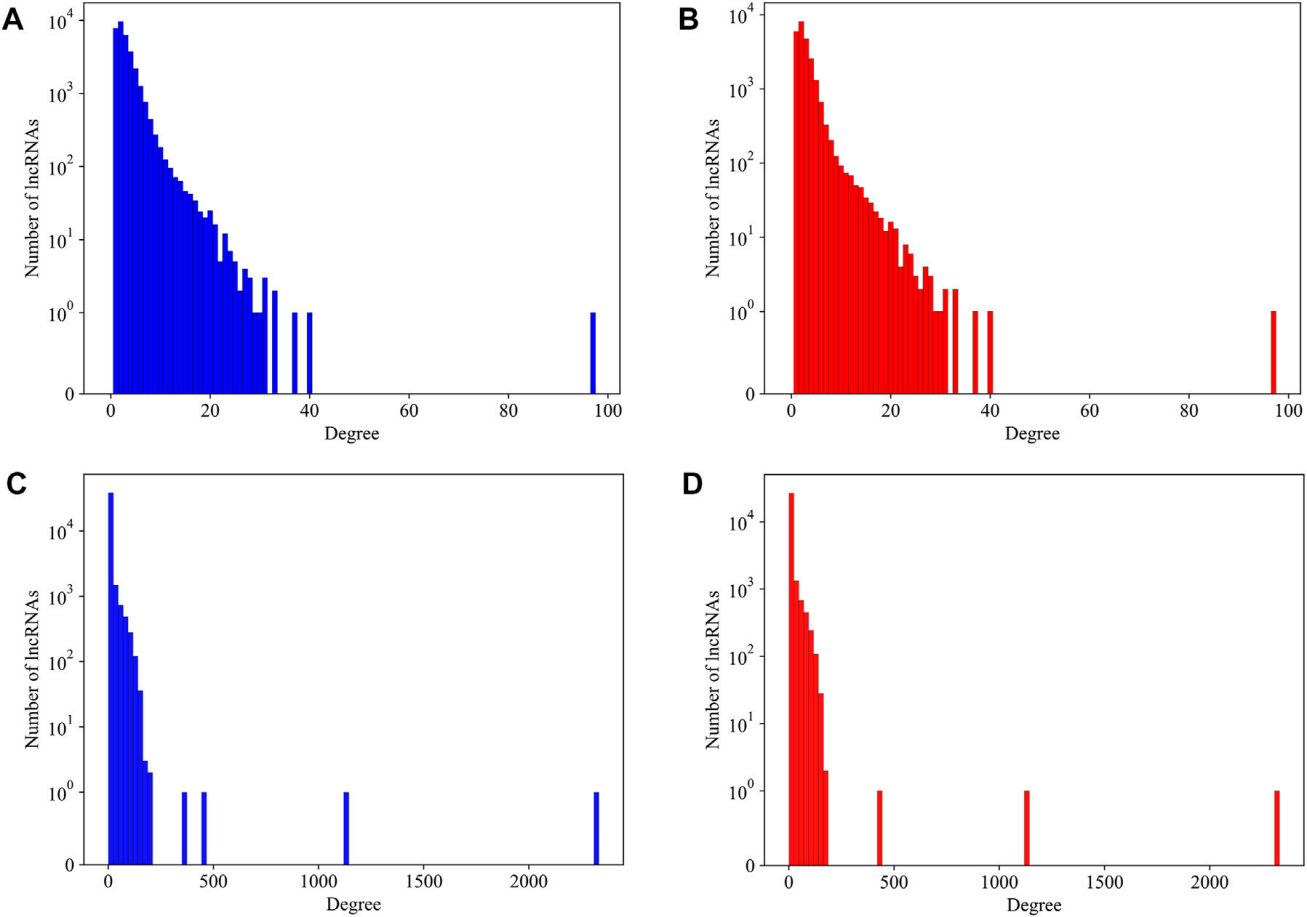
The degree distribution of lncRNAs in mouse network and human network with and without over-length lncRNAs. **(A)** The degree distribution of lncRNAs in mouse network with over-length lncRNAs; **(B)** The degree distribution of lncRNAs in mouse network without over-length lncRNAs; **(C)** The degree distribution of lncRNAs in human network with over-length lncRNAs; **(D)** The degree distribution of lncRNAs in human network without over-length lncRNAs.

As we have mentioned in the method section, several lncRNAs with a length too long to calculate its secondary structure were not counted in our analysis. It becomes a question whether these lncRNAs have preferences to large or small amounts of interactions. We plot the degree distribution with and without those over-length lncRNAs for mouse and human datasets, respectively, in Figure 4. It is hard to find differences on the degree distributions. We therefore believe that, for a lncRNA, its length alone is not a major contributing factor to its interactions in the LPPI network. This also implied that the essentiality, which we believe to be associated with local network structure, has no direct relationship with the length of the lncRNA. These over-length lncRNAs were kept in the network as dummy nodes, which means we did not compute their essentiality at all, regardless of whether they have a degree over the threshold or not.

### Integrating Centrality Measures and the GIC Score

**FIGURE 5:**
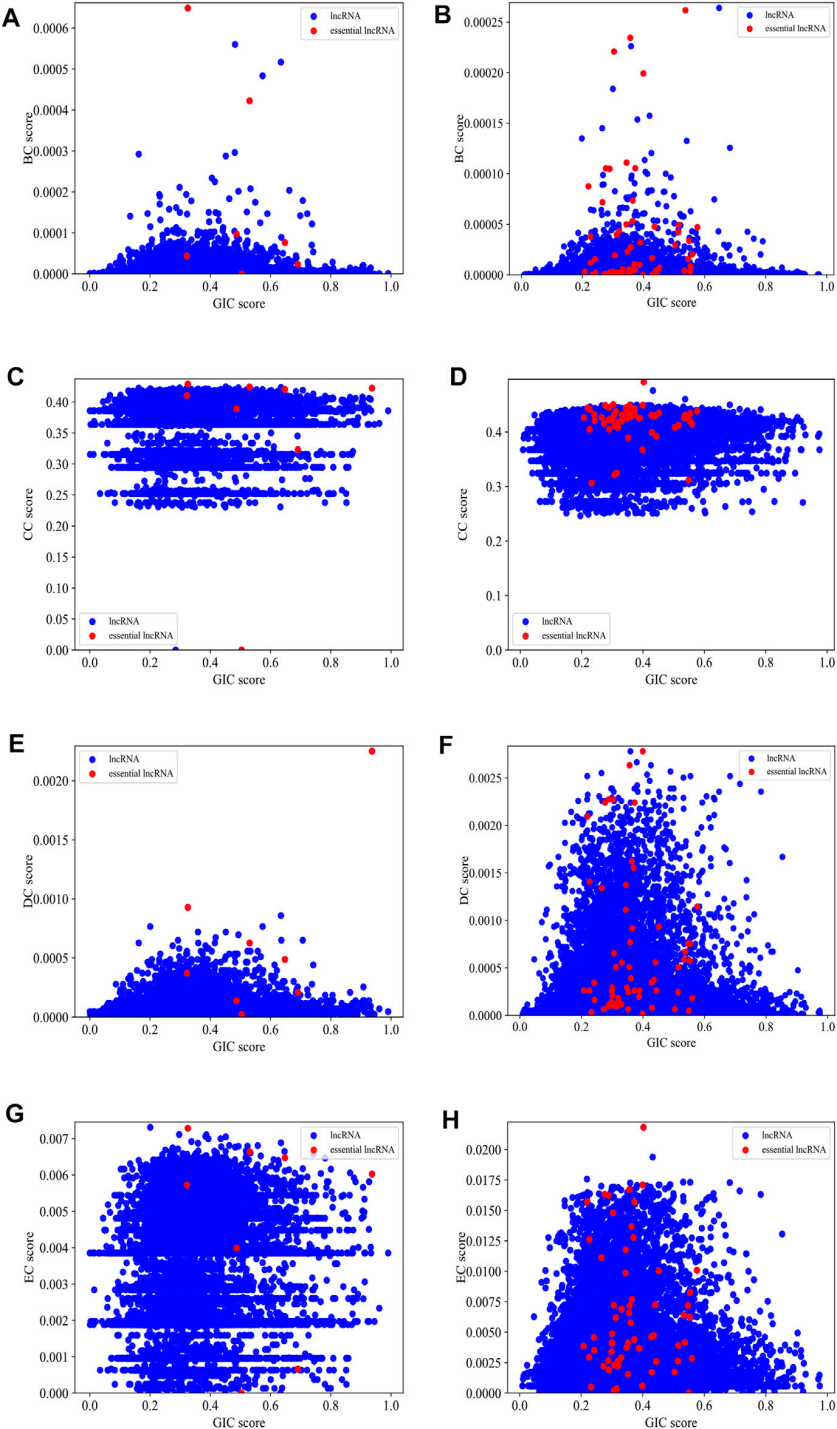
The scatter plots of GIC pairing with each of the four types of centralities on mouse dataset and human dataset, respectively. **(A)** BC pairing with GIC on mouse dataset; **(B)** BC pairing with GIC on human dataset; **(C)** CC pairing with GIC on mouse dataset; **(D)** CC pairing with GIC on human dataset; **(E)** DC pairing with GIC on mouse dataset; **(F)** DC pairing with GIC on human dataset; **(G)** EC pairing with GIC on mouse dataset; **(H)** EC pairing with GIC on human dataset. Red dots represent known essential lncRNAs, while blue dots represented all others. When drawing panel (A), BC scores of mouse *HOTAIR* and *Xist* are too high to be plotted in the scope. Their (BC,GIC) values are (0.01.0.39) and (0.01.0.94). When drawing panel (B), *NEAT1*, *MALAT1*, *U1* are too distant to other dots, so they cannot be reasonably plotted in the scope. Their (BC,GIC) values are (0.03.0.40), (0.01.0.43) and (0.005.0.54).

Figure 5 gives scatter plots of GIC pairing with each of the four types of centralities on human and mouse datasets, respectively. For the mouse dataset, the red dots, which represent essential lncRNAs, tend to appear in the top-right part of the plots, while the blue dots, which denote all other lncRNAs, spread much wider. Although the red dots are relatively rare, but their top-right preference is still observable. For human dataset, this preference is not intuitively obvious.

This allows us to carry out further quantitative analysis on combining the centrality measures and the GIC scores. A primary challenge is that the number of known essential lncRNAs is too small for a machine learning algorithm to train on. In addition, some essential lncRNAs are only involved in a very limited number of interactions. For example, the *Braveheart* (Bvht) lncRNA, which is essential, has only one interaction record in the database. We think this may be due to the incomprehensive knowledge of the lncRNA-protein interaction network. As the estimation of centrality measures highly rely on the interaction enrichment of a node in the network, when dealing with a lncRNA with limited number of interactions, we turn to rely on the GIC score.

With the settings in the method section, we combined four types of centrality measures and the GIC scores. On the mouse dataset, we identified 2284 essential lncRNAs from altogether 24450 lncRNAs. Among the 2284 lncRNAs, eight lncRNAs are known to be essential, accounting for 100% of all known essential lncRNAs, resulting a *p*-value = 5.73 × 10^–9^ (Fisher’s exact test). On the human dataset, we identified 5063 essential lncRNAs, from altogether 29481 lncRNAs, Among the 5063 essential lncRNAs, 41 lncRNAs are reported to be essential in various conditions in literatures, accounting for 65% of all curated essential lncRNAs (*p*-value = 3.59 × 10^–17^, Fisher’s exact test). This result clearly indicates that our method is effective to identify essential lncRNAs.

### Systematic Comparison Between Different Configurations of SGII

**FIGURE 6:**
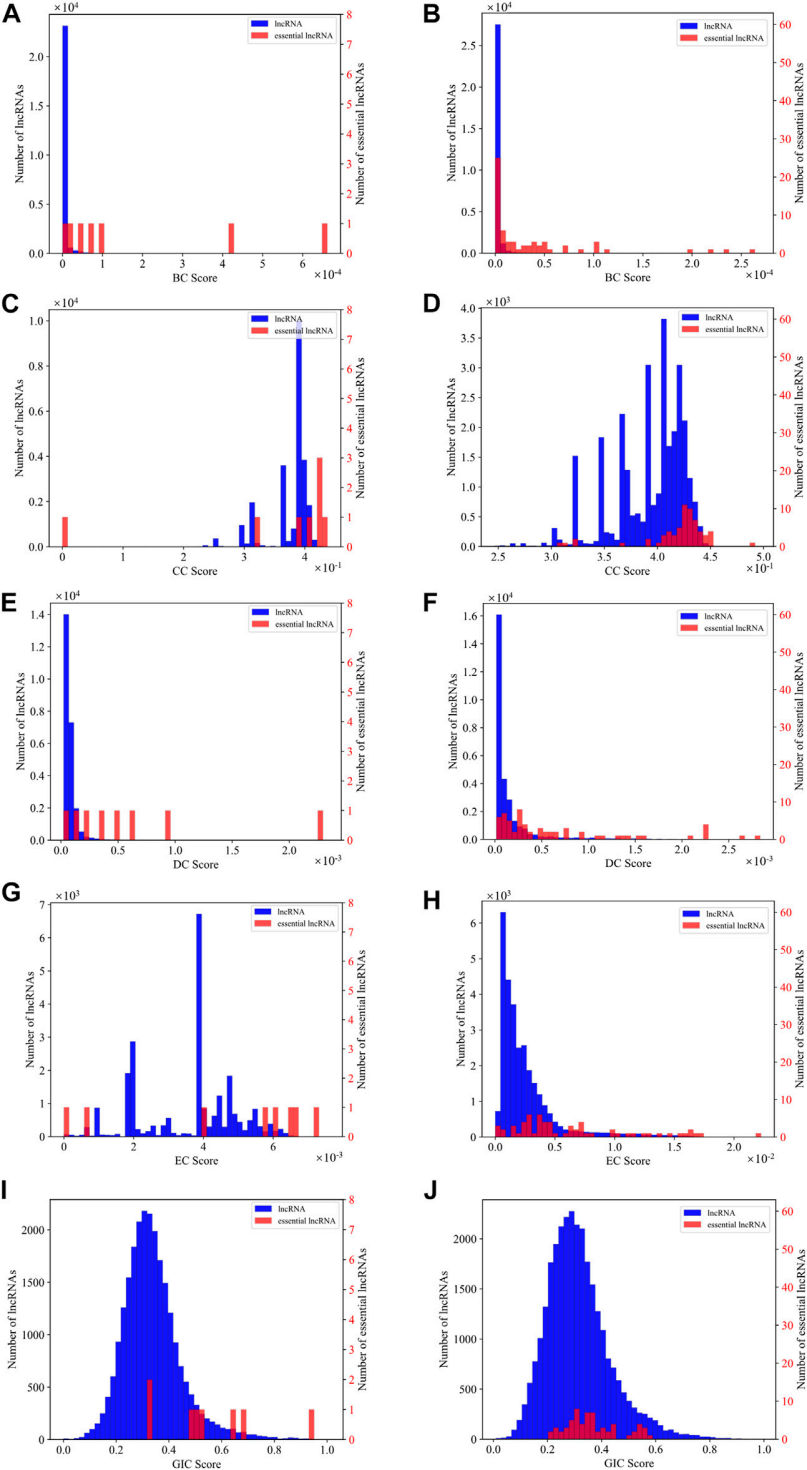
The distribution density of different centralities and the GIC scores on mouse dataset and human dataset respectively. **(A)** The distribution density of BC on mouse dataset; **(B)** The distribution density of BC on human dataset; **(C)** The distribution density of CC on mouse dataset; **(D)** The distribution density of CC on human dataset; **(E)** The distribution density of DC on mouse dataset; **(F)** The distribution density of DC on human dataset; **(G)** The distribution density of EC on mouse dataset; **(H)** The distribution density of EC on human dataset; **(I)** The distribution density of GIC on mouse dataset; **(J)** The distribution density of GIC on human dataset. The red bars represent known essential lncRNAs, while the blue bars for all others. The vertical axis for the red bars are on the right side of the panel, while blue on left. When drawing panel (A), BC scores of mouse *HOTAIR* and *Xist* are too high to be plotted in the scope. Their BC values are 0.01 and 0.01. When drawing panel (B), BC scores of human *NEAT1*, *MALAT1*, *U1* are too far to be drawn in the scope. Their BC values are 0.03, 0.01 and 0.005.

As SGII is the first attempt to combine the network information and sequence information to identify essential lncRNAs, we explore which kind of centrality measure is more capable to identify essential lncRNAs along with the GIC scores. We first plot the distribution density of different centralities and the GIC scores on mouse and human datasets respectively. As in Figure 6, BC and DC centrality measures along with the GIC scores appear to have much better separation than the CC and EC measures on the mouse dataset, while on the human dataset, only BC and DC present an intuitive separation.

However, considering the large differences on axis scale for essential lncRNAs and all lncRNAs, these intuitive observations may be misleading. Therefore, we performed a quantitative comparison using eight different conditions, GIC alone, GIC combined with each one of four types of centralities, GIC combined with BC and DC, GIC combined with CC and EC, and GIC combined with all four types of centralities. The parameters of all comparison are optimized as in method section (Table 1).

**TABLE 1:** Comparison for different configurations of SGII on mouse and human datasets.

Methods	Dataset	Sen^a^ (%)	FPR^b^ (%)	Fisher’s exact test score^c^
GIC^d^	Mouse	75.00	8.98	4.90
BC + GIC	Mouse	100.00	9.53	8.16
CC + GIC	Mouse	100.00	9.48	8.18
DC + GIC	Mouse	100.00	9.55	8.16
EC + GIC	Mouse	100.00	9.32	8.24
BC + DC + GIC	Mouse	87.50	4.54	8.50
CC + EC + GIC	Mouse	100.00	9.31	8.24
BC + CC + DC + EC + GIC	Mouse	100.00	9.31	8.24
GIC	Human	26.98	14.97	1.91
BC + GIC	Human	66.67	12.49	22.61
CC + GIC	Human	63.49	16.37	16.15
DC + GIC	Human	71.43	20.51	17.25
EC + GIC	Human	66.67	19.94	14.90
BC + DC + GIC	Human	71.43	18.33	19.23
CC + EC + GIC	Human	65.08	18.19	15.45
BC + CC + DC + EC + GIC	Human	65.08	17.07	16.45

a Sen stands for Sensitivity, as Eq. 17.

b FPR, stands for False Positive Rate, as Eq. 18.

c Fisher’s Exact Test Score is defined in Eq. 19.

d When GIC, was used alone, it is applied on all lncRNAs.

The first observation on Table 1 is that the best combination of centrality measure and the GIC is not the combination of all four types of centralities. For the mouse dataset, the BC + DC + GIC method has the best significance level and lowest FPR value. For the human dataset, the BC + GIC method reaches the highest significance level. A second to the best significance level is obtained again by BC + DC + GIC method, with the highest sensitivity value. Therefore, we think that the BC + DC + GIC may be a better way to identify essential lncRNAs than the current configuration of SGII. This consists with the impression from Figure 6. However, due to the limited number of available data and current results, it is possible that this observation does not reflect a comprehensive scene of identifying essential lncRNAs. Therefore, we keep the configuration of SGII to combine all four kinds of centralities and the GIC score, for an unbiased way of identifying essential lncRNAs.

### Comparative Analysis Between Human and Mouse Essential lncRNAs

At a closer look to Table 1, it appears that the Fisher’s exact test reports much more significant results on both datasets when GIC is combined with centralities, which proves that integration of centrality measures and GIC is effective. Another observation is that SGII gives under-expected sensitivity values on the human dataset. However, the significance levels on the human dataset are generally way higher than that of the mouse dataset. This may be the results of two differences between the mouse and the human datasets. First, the human dataset is collected from literatures of lncRNAs in various conditions, including tumor cell line experiments. Essential lncRNAs, which are identified by one type of cell line experiments, may be different to those from the original essential gene definitions. As direct essential gene experiments on human are not feasible, the quality of the dataset is not comparable to the mouse dataset. This also applies to the coding gene data (Austin et al., 2004). Secondly, the number of essential lncRNAs in the human dataset is roughly eight times of that of mouse dataset. Since the computation process of the significance level is affected by the raw counts, it is anticipated that systematic differences on significance levels exist.

To further confirm the above explanations, we performed the following analysis. We find homologous genes of human essential lncRNAs in mouse. According to the studies in coding genes, these genes are likely to also produce essential lncRNAs(Georgi et al., 2013). Altogether 11 homologous genes in mouse were identified as lncRNA genes in the mouse LPPI network. We used SGII to test if we can identify these homolog essential lncRNAs (Table 2).

**TABLE 2:** Performance analysis on mouse homologs to human essential lncRNAs.

Methods	Sen^a^ (%)	FPR^b^ (%)	Fisher’s exact test score^c^
GIC^d^	45.45	8.98	2.77
BC + GIC	72.73	1.79	11.75
CC + GIC	45.45	1.22	6.90
DC + GIC	54.55	1.25	8.75
EC + GIC	45.45	1.17	7.00
BC + DC + GIC	81.82	5.89	9.36
CC + EC + GIC	45.45	1.17	7.00
BC + CC + DC + EC + GIC	45.45	1.17	7.00

a Sen stands for Sensitivity, as Eq. 17.

b FPR, stands for False Positive Rate, as Eq. 18.

c Fisher’s Exact Test Score is defined in Eq. 19.

d When GIC, was used alone, it is applied on all lncRNAs.

Obviously, sensitivity is dropping in comparison to the mouse essential lncRNAs. However, it should be noted that the FPR is also dropping, which indicates much less false positives. The significance levels remain almost the same as the mouse essential lncRNAs. Again, the BC + GIC method obtained the best significance level, while the BC + DC + GIC method obtained a second to the best significance level with the highest sensitivity. This result confirmed that the significance level difference between human and mouse dataset is largely caused by the raw counts of the dataset. It also suggests that the BC + GIC or BC + DC + GIC method may be a better choice than combining all types of centralities and the GIC score.

The importance of BC can be understood intuitively. If we think the cellular system as a system composed of molecules. The interactions between molecules transfer information. A high BC value indicated that the node is critical as an information hub in many shortest paths between other nodes. Therefore, dropping such nodes will easily break many information channels simultaneously, which will eventually destroy the whole system. That makes it an essential node in the network.

For the DC measure, the intrinsic mechanism is similar. The DC measure is directly associated to the degree of a node. If a node with many edges is dropped, it is more likely that the whole network collapses. This consists with the observations in coding genes. In addition, although some other kinds of centralities, like the NC (new centrality) (Wang et al., 2012), can identify essential coding genes better, it does not work well in non-coding genes. This is an expected result. For NC to work in the LPPI network, it requires that dense interactions exist among the proteins that interacting the same lncRNAs. However, we did not observe this phenomenon in our dataset. The NC is difficult to be estimated for many lncRNAs, due to lacking such kind of interactions.

### Functional Analysis of Essential lncRNA in the Mouse Genome

We took the essential lncRNA gene in mouse genome for functional analysis. For every lncRNA that was predicted as essential in mouse genome, we first map this lncRNA to the Ensembl database (Howe et al., 2021) using either gene name or sequence information. The mapped genes are then uploaded to the Gene Ontology online system for functional enrichment analysis. The top three enrichment of functions are “nucleic acid binding” (GO:0003676), “heterocyclic compound binding” (GO:1901363) and “organic cyclic compound binding” (GO:0097159). As we have mentioned, this is expected for lncRNAs. They realize their functions through bindings with other molecules.

## Conclusion

SGII is the first attempt to combine lncRNA-protein interactions and lncRNA sequence information for identifying essential non-coding RNAs. Since the study on collecting and identifying essential coding genes has been performed for over a decade, it is time to step forward to the essentiality of non-coding genes, as non-coding genes are much more common than coding genes in mouse and human genomes. Due to the limited number of known essential lncRNAs, SGII does not use conventional machine learning algorithms, but applies simple scoring schemes and statistical tests. By combining BC, CC, DC, EC and GIC scores, SGII achieved a better performance than using only sequence information. Since the knowledge for constructing LPPI network may be incomprehensive, we applied the centrality measures only on those lncRNAs with enough interactions. For those lncRNAs with limited number of interactions, we turned to rely on its sequence to score the essentiality.

The results support our assumption that essential lncRNAs have similar roles as essential coding genes in the LPPI network. Particularly, we found that BC appears to be more important than other kinds of centrality measures. Due to the limited number of known essential lncRNAs, it is not feasible to explore further optimization of different weight on different centralities. When more essential lncRNAs are reported and recorded, we believe that modern machine learning algorithms will provide deeper insights in identifying essential non-coding genes. As a summary, we listed the prediction results of SGII on mouse and human datasets in Supplementary Table S3 in supplementary materials, which may be useful for life science studies. A more comprehensive collection of essential lncRNAs is being curated. We plan to establish a database that is dedicated in recording essential lncRNA information in future.

## Data Availability

The original contributions presented in the study are included in the article/Supplementary Material, further inquiries can be directed to the corresponding authors.

## References

[B1] Austin C. P. Battey J. F. Bradley A. Bucan M. Capecchi M. Collins F. S. (2004). The Knockout Mouse Project. Nat. Genet. 36, 921–924. 10.1038/ng0904-921 15340423 PMC2716027

[B2] Campos T. L. Korhonen P. K. Gasser R. B. Young N. D. (2019). An Evaluation of Machine Learning Approaches for the Prediction of Essential Genes in Eukaryotes Using Protein Sequence-Derived Features. Comput. Struct. Biotechnol. J. 17, 785–796. 10.1016/j.csbj.2019.05.008 31312416 PMC6607062

[B3] Chen L.-L. (2016). Linking Long Noncoding RNA Localization and Function. Trends Biochem. Sci. 41, 761–772. 10.1016/j.tibs.2016.07.003 27499234

[B4] Da Sacco L. Baldassarre A. Masotti A. (2012). Bioinformatics Tools and Novel Challenges in Long Non-coding RNAs (lncRNAs) Functional Analysis. Ijms 13, 97–114. 10.3390/ijms13010097 22312241 PMC3269675

[B5] Fenoglio C. Ridolfi E. Galimberti D. Scarpini E. (2013). An Emerging Role for Long Non-coding RNA Dysregulation in Neurological Disorders. Ijms 14, 20427–20442. 10.3390/ijms141020427 24129177 PMC3821623

[B6] Georgi B. Voight B. F. Bućan M. (2013). From Mouse to Human: Evolutionary Genomics Analysis of Human Orthologs of Essential Genes. Plos Genet. 9, e1003484. 10.1371/journal.pgen.1003484 23675308 PMC3649967

[B7] Grote P. Wittler L. Hendrix D. Koch F. Währisch S. Beisaw A. (2013). The Tissue-specific lncRNA Fendrr Is an Essential Regulator of Heart and Body wall Development in the Mouse. Dev. Cel. 24, 206–214. 10.1016/j.devcel.2012.12.012 PMC4149175 23369715

[B8] Hao Y. Wu W. Li H. Yuan J. Luo J. Zhao Y. (2016). NPInter v3.0: an Upgraded Database of Noncoding RNA-Associated Interactions. Database 2016, baw057. 10.1093/database/baw057 27087310 PMC4834207

[B9] Howe K. L. Achuthan P. Allen J. Allen J. Alvarez-Jarreta J. Amode M. R. (2021). Ensembl 2021. Nucleic Acids Res. 49, D884–D891. 10.1093/nar/gkaa942 33137190 PMC7778975

[B10] Hu H. Zhu C. Ai H. Zhang L. Zhao J. Zhao Q. (2017). LPI-ETSLP: lncRNA-Protein Interaction Prediction Using Eigenvalue Transformation-Based Semi-supervised Link Prediction. Mol. Biosyst. 13, 1781–1787. 10.1039/c7mb00290d 28702594

[B11] Jathar S. Kumar V. Srivastava J. Tripathi V. (2017). Technological Developments in lncRNA Biology. Adv. Exp. Med. Biol. 1008, 283–323. 10.1007/978-981-10-5203-3_10 28815544

[B12] Jeong H. Mason S. P. Barabási A.-L. Oltvai Z. N. (2001). Lethality and Centrality in Protein Networks. Nature 411, 41–42. 10.1038/35075138 11333967

[B13] Joy M. P. Brock A. Ingber D. E. Huang S. (2005). High-betweenness Proteins in the Yeast Protein Interaction Network. J. Biomed. Biotechnol. 2005, 96–103. 10.1155/JBB.2005.96 16046814 PMC1184047

[B14] Khalil A. M. Rinn J. L. (2011). RNA-protein Interactions in Human Health and Disease. Semin. Cel Dev. Biol. 22, 359–365. 10.1016/j.semcdb.2011.02.016 PMC3184770 21333748

[B15] Klattenhoff C. A. Scheuermann J. C. Surface L. E. Bradley R. K. Fields P. A. Steinhauser M. L. (2013). Braveheart, a Long Noncoding RNA Required for Cardiovascular Lineage Commitment. Cell 152, 570–583. 10.1016/j.cell.2013.01.003 23352431 PMC3563769

[B16] Lee J. T. (2000). Disruption of Imprinted X Inactivation by Parent-Of-Origin Effects at Tsix. Cell 103, 17–27. 10.1016/s0092-8674(00)00101-x 11051544

[B17] Li L. Chang H. Y. (2014). Physiological Roles of Long Noncoding RNAs: Insight from Knockout Mice. Trends Cel Biol. 24, 594–602. 10.1016/j.tcb.2014.06.003 PMC4177945 25022466

[B18] Li M. Zhang H. Wang J.-x. Pan Y. (2012). A New Essential Protein Discovery Method Based on the Integration of Protein-Protein Interaction and Gene Expression Data. BMC Syst. Biol. 6, 15. 10.1186/1752-0509-6-15 22405054 PMC3325894

[B19] Li Y. Egranov S. D. Yang L. Lin C. (2019). Molecular Mechanisms of Long Noncoding RNAs‐mediated Cancer Metastasis. Genes Chromosomes Cancer 58, 200–207. 10.1002/gcc.22691 30350428 PMC10642708

[B20] Liu W. Jiang Y. Peng L. Sun X. Gan W. Zhao Q. (2021). Inferring Gene Regulatory Networks Using the Improved Markov Blanket Discovery Algorithm. Interdiscip. Sci. Comput. Life Sci. 10.1007/s12539-021-00478-9 34495484

[B21] Lorenz R. Bernhart S. H. Höner zu Siederdissen C. Tafer H. Flamm C. Stadler P. F. (2011). ViennaRNA Package 2.0. Algorithms Mol. Biol. 6, 26. 10.1186/1748-7188-6-26 22115189 PMC3319429

[B22] Marahrens Y. Panning B. Dausman J. Strauss W. Jaenisch R. (1997). Xist-deficient Mice Are Defective in Dosage Compensation but Not Spermatogenesis. Genes Dev. 11, 156–166. 10.1101/gad.11.2.156 9009199

[B23] Mercer T. R. Dinger M. E. Mattick J. S. (2009). Long Non-coding RNAs: Insights into Functions. Nat. Rev. Genet. 10, 155–159. 10.1038/nrg2521 19188922

[B24] Oughtred R. Rust J. Chang C. Breitkreutz B. J. Stark C. Willems A. (2021). TheBioGRIDdatabase: A Comprehensive Biomedical Resource of Curated Protein, Genetic, and Chemical Interactions. Protein Sci. 30, 187–200. 10.1002/pro.3978 33070389 PMC7737760

[B25] Penny G. D. Kay G. F. Sheardown S. A. Rastan S. Brockdorff N. (1996). Requirement for Xist in X Chromosome Inactivation. Nature 379, 131–137. 10.1038/379131a0 8538762

[B26] Pyfrom S. C. Luo H. Payton J. E. (2019). PLAIDOH: a Novel Method for Functional Prediction of Long Non-coding RNAs Identifies Cancer-specific LncRNA Activities. BMC Genomics 20, 137. 10.1186/s12864-019-5497-4 30767760 PMC6377765

[B27] Rinn J. L. Chang H. Y. (2012). Genome Regulation by Long Noncoding RNAs. Annu. Rev. Biochem. 81, 145–166. 10.1146/annurev-biochem-051410-092902 22663078 PMC3858397

[B28] Sado T. Wang Z. Sasaki H. Li E. (2001). Regulation of Imprinted X-Chromosome Inactivation in Mice by Tsix. Development 128, 1275–1286. 10.1242/dev.128.8.1275 11262229

[B29] Sauvageau M. Goff L. A. Lodato S. Bonev B. Groff A. F. Gerhardinger C. (2013). Multiple Knockout Mouse Models Reveal lincRNAs Are Required for Life and Brain Development. Elife 2, e01749. 10.7554/eLife.01749 24381249 PMC3874104

[B30] Schmitt A. M. Chang H. Y. (2016). Long Noncoding RNAs in Cancer Pathways. Cancer Cell 29, 452–463. 10.1016/j.ccell.2016.03.010 27070700 PMC4831138

[B31] Uchida S. Dimmeler S. (2015). Long Noncoding RNAs in Cardiovascular Diseases. Circ. Res. 116, 737–750. 10.1161/CIRCRESAHA.116.302521 25677520

[B32] Wang J. Min Li M. Huan Wang H. Yi Pan Y. (2012). Identification of Essential Proteins Based on Edge Clustering Coefficient. Ieee/acm Trans. Comput. Biol. Bioinf. 9, 1070–1080. 10.1109/TCBB.2011.147 22084147

[B33] Wang J. Peng W. Wu F.-X. (2013). Computational Approaches to Predicting Essential Proteins: a Survey. Proteomices. Clin. Appl. 7, 181–192. 10.1002/prca.201200068 23165920

[B34] Watanabe T. Sato T. Amano T. Kawamura Y. Kawamura N. Kawaguchi H. (2008). Dnm3os, a Non-coding RNA, Is Required for normal Growth and Skeletal Development in Mice. Dev. Dyn. 237, 3738–3748. 10.1002/dvdy.21787 18985749

[B35] Wuchty S. Stadler P. F. (2003). Centers of Complex Networks. J. Theor. Biol. 223, 45–53. 10.1016/S0022-5193(03)00071-7 12782116

[B36] Yildirim E. Kirby J. E. Brown D. E. Mercier F. E. Sadreyev R. I. Scadden D. T. (2013). Xist RNA Is a Potent Suppressor of Hematologic Cancer in Mice. Cell 152, 727–742. 10.1016/j.cell.2013.01.034 23415223 PMC3875356

[B37] Zeng P. Chen J. Meng Y. Zhou Y. Yang J. Cui Q. (2018). Defining Essentiality Score of Protein-Coding Genes and Long Noncoding RNAs. Front. Genet. 9, 380. 10.3389/fgene.2018.00380 30356729 PMC6189311

[B38] Zhang R. Ou H.-Y. Zhang C.-T. (2004). DEG: a Database of Essential Genes. Nucleic Acids Res. 32, 271D–272D. 10.1093/nar/gkh024 14681410 PMC308758

[B39] Zhang W. Yue X. Tang G. Wu W. Huang F. Zhang X. (2018). SFPEL-LPI: Sequence-Based Feature Projection Ensemble Learning for Predicting LncRNA-Protein Interactions. Plos Comput. Biol. 14, e1006616. 10.1371/journal.pcbi.1006616 30533006 PMC6331124

[B40] Zhang Z. Luo Y. Hu S. Li X. Wang L. Zhao B. (2020). A Novel Method to Predict Essential Proteins Based on Tensor and HITS Algorithm. Hum. Genomics 14, 14. 10.1186/s40246-020-00263-7 32252824 PMC7137323

[B41] Zhang L. Yang P. Feng H. Zhao Q. Liu H. (2021). Using Network Distance Analysis to Predict lncRNA-miRNA Interactions. Interdiscip. Sci. Comput. Life Sci. 13, 535–545. 10.1007/s12539-021-00458-z 34232474

[B42] Zhao Y. Li H. Fang S. Kang Y. Wu W. Hao Y. (2016). NONCODE 2016: an Informative and Valuable Data Source of Long Non-coding RNAs. Nucleic Acids Res. 44, D203–D208. 10.1093/nar/gkv1252 26586799 PMC4702886

[B43] Zhao Y. Teng H. Yao F. Yap S. Sun Y. Ma L. (2020). Challenges and Strategies in Ascribing Functions to Long Noncoding RNAs. Cancers 12, 1458. 10.3390/cancers12061458 PMC7352683 32503290

[B44] Zhong J. Wang J. Peng W. Zhang Z. Pan Y. (2013). Prediction of Essential Proteins Based on Gene Expression Programming. BMC Genomics 14 Suppl 4, S7. 10.1186/1471-2164-14-S4-S7 PMC3856491 24267033

[B45] Zhou Y. Zhang X. Klibanski A. (2012). MEG3 Noncoding RNA: a Tumor Suppressor. J. Mol. Endocrinol. 48, R45–R53. 10.1530/JME-12-0008 22393162 PMC3738193

[B46] Zhu J. Fu H. Wu Y. Zheng X. (2013). Function of lncRNAs and Approaches to lncRNA-Protein Interactions. Sci. China Life Sci. 56, 876–885. 10.1007/s11427-013-4553-6 24091684

